# Intervertebral-spreader-assisted anterior cervical discectomy and fusion prevents postoperative axial pain by alleviating facet joint pressure

**DOI:** 10.1186/s13018-022-02983-z

**Published:** 2022-02-15

**Authors:** Chen Xu, Ruizhe Wang, Jingchi Li, Huajian Zhong, Zifang Zhang, Cheng Cui, Baifeng Sun, Ye Tian, Huajiang Chen, Xiaolong Shen, Yang Liu, Wen Yuan

**Affiliations:** 1 Spine Center, Department of Orthopedics, Shanghai Changzheng Hospital, Naval Medical University, 415th Fengyang Road, Shanghai, 200003 People’s Republic of China; 2 Department of Orthopedics, Hospital (T.C.M) Affiliated to Southwest Medical University, 182th Chunhui Road, Luzhou, Sichuan Province, 646000 People’s Republic of China

**Keywords:** Vertebral spreader, Degenerative cervical spondylosis, Facet joint, Axial pain

## Abstract

**Objective:**

To evaluate the relationship of postoperative cervical axial pain with different vertebral distraction methods used during ACDF procedures in cervical spondylosis patients.

**Methods:**

Ninety-four single-level cervical spondylotic myelopathy patients with significantly loss of intervertebral disc height who underwent ACDF surgery in our institute between January 2018 and January 2020 were enrolled. Cervical spine lateral radiographs were taken preoperatively, 3 days, 1-month, 2-month and 6-month after the surgery. The intervertebral disc height (IDH), interfacet distance (IFD), JOA (Japanese Orthopaedic Association) score, NDI (Neck Disability Index) score, nVAS (Neck Visual Analogue Scale) score and aVAS (Arm Visual Analogue Scale) score were measured. The correlation of clinical parameters and intervertebral disc height was evaluated. Then the correlation of clinical outcomes and different distraction method was evaluated. The patients were randomly divided into two groups, one uses Casper pin distractor system alone for distraction (Caspar alone group) and the other uses spreader assisted distraction method (Casper + spreader group). In biomechanical study, four cervical spine cadavers were selected for facet pressure measurements under different vertebral distraction methods, and the facet joint pressure was measured using force sensors.

**Results:**

Satisfactory cervical fusion and neurological recovery were achieved in all patients. No significant correlation of IDH, IFD, JOA, NDI or aVAS with nVAS score was found. No significant difference between the change in disc height and clinical outcomes was found. However, by comparing the clinical parameters of patients in different vertebral distraction groups, we found significant changes in the early nVAS and NDI scores (*P* = 0.11, *P* = 0.48) of the Casper + spreader group (3 days postoperation), and was associated with a better nVAS score at 2 months postoperation (*P* < 0.05). The biomechanical study in cervical cadavers also showed significantly and continuously decreased facet joint pressure in the spreader assisted vertebral distraction group (*P* < 0.01).

**Conclusions:**

Spreader-assisted vertebral distraction method effectively alleviates postoperative neck pain in degenerative cervical spondylosis patients treated with ACDF. The mechanism may be related to the transient relief of facet joint pressure during the vertebral distraction procedure in ACDF.

## Introduction

Anterior cervical discectomy and fusion (ACDF), as the most important and reliable anterior approach to treat cervical spondylosis, was first described in 1957 [1–3]. Now, it is widely used all over the world [4, 5]. This technique requires sufficient exposure and decompression [6]. Compared to the posterior approach, the anterior approach has many advantages, such as complete removal of the disc and osteophytes, a small surgical incision and less surgical trauma [7]. However, patients undergoing ACDF still develop some complications, such as postoperative axial pain, pseudarthrosis, and adjacent-level disc degeneration [8–10]. Among these complications, recent studies have indicated that postoperative axial pain, a complication that has long been thought to be related to posterior surgical approaches, has become more prominent and was observed in 38.3% of patients [11]. The influence of disc space enlargement, cervical range of motion, cervical curvature and the pressure of the facet joint were all possible risk factors for postoperative axial pain according to current studies [12, 13].

During the standard ACDF procedure, vertebral distraction is routinely used to obtain fine surgical exposure and promote the implantation of intervertebral spacers or cages [14]. However, overdistraction will cause excess load on the facet joint and injury to soft tissue, and underdistraction will result in insufficient exposure and decompression [15, 16]. It is thought that overdistraction may be the cause of postoperative axial pain, but there have been controversies over the mechanism by which this occurs. Recent studies have shown that the vertebral distractors used during vertebral distraction may place abnormal loads on the facet joint [17] and pointed out that this will directly lead to worse Visual Analogue Scale (VAS) scores and neck disability indices (NDIs), which are related to postoperative axial pain [18, 19]. In our patients, compared to the use of vertebral distractors, we proposed that the use of vertebral spreaders in vertebral distraction may decrease the incidence of postoperative axial pain related to the ACDF procedure. We assumed that posterior vertebral distraction using a vertebral spreader may protect against postoperative neck pain by alleviating the pressure on the facet joint. To verify this hypothesis, we selected patients in whom either vertebral distractors alone or vertebral-spreader-assisted vertebral distraction was used and reviewed their clinical parameters to compare their postoperative outcomes. Furthermore, we performed cadaver biomechanical studies to assess the function of vertebral-spreader-assisted vertebral distraction on facet joint pressure.

## Materials and methods

### Patients

All study procedures were approved by the institute chancellor’s Human Research Committee in accordance with protocol 2015-0018. The cases of patients who underwent single-level anterior cervical discectomy and fusion (ACDF) for diagnosed cervical radiculopathy or myelopathy between January 2018 and January 2020 in our institute were enrolled. The inclusion criteria were (1) single-level cervical spondylotic myelopathy or radiculopathy resulting from a herniated intervertebral disc confirmed by MRI and CT scans, (2) ACDF occurring in the subaxial cervical spine, (3) more than 3 months of conservative treatment before admission to the hospital for surgery and (4) the intervertebral disc height of the responsible segment is significantly narrowed (loss of disc height of 50% or more relative to the above normal disc). The exclusion criteria were (1) a previous history of cervical spine surgery; (2) ACDF associated with cervical stenosis or cervical deformity resulting from spinal injury, tumour, infection, congenital disorders, OPLL or inflammatory arthritis (including ankylosing spondylitis and rheumatoid arthritis); and (3) Patients with neck and back pain due to inflammatory causes, visceral origin, systemic infections affecting spine, metabolic bone diseases, fractures in the vertebral column, past surgeries in the spine, and spinal tumours. Informed consent was confirmed, and each patient is blinded and randomly enrolled into the two surgical groups using the random number tables method. All patients enrolled must finish at least 1 year of follow-up to be included in this study, and a total of 94 patients (40 men and 54 women, with a mean age of 57.6 years, ranging from 40 to 75 years) passed the criteria.

### Clinical and radiological assessment

Clinical assessment was performed by a spine specialist who was blinded to the patients’ information. The patients were asked to check their neck disability index (NDI) and grade their Japanese Orthopaedic Association (JOA) scores and neck and arm pain intensity before surgery and at routine postoperative intervals of 3 days and 1, 2, and 6 months. The NDI scores were expressed in a range from 0 (no disability) to 50 (maximum disability). Pain intensity was reported on a scale of 0 to 10 using the subjective visual analogue scale (VAS; 0 = no pain; 10 = the worst pain imaginable). During the follow-up period, the incidence and degree of neck pain (neck VAS score) were routinely checked, and any increment in the neck VAS score after the surgery was defined as postoperative axial pain.

**Fig. 1:**
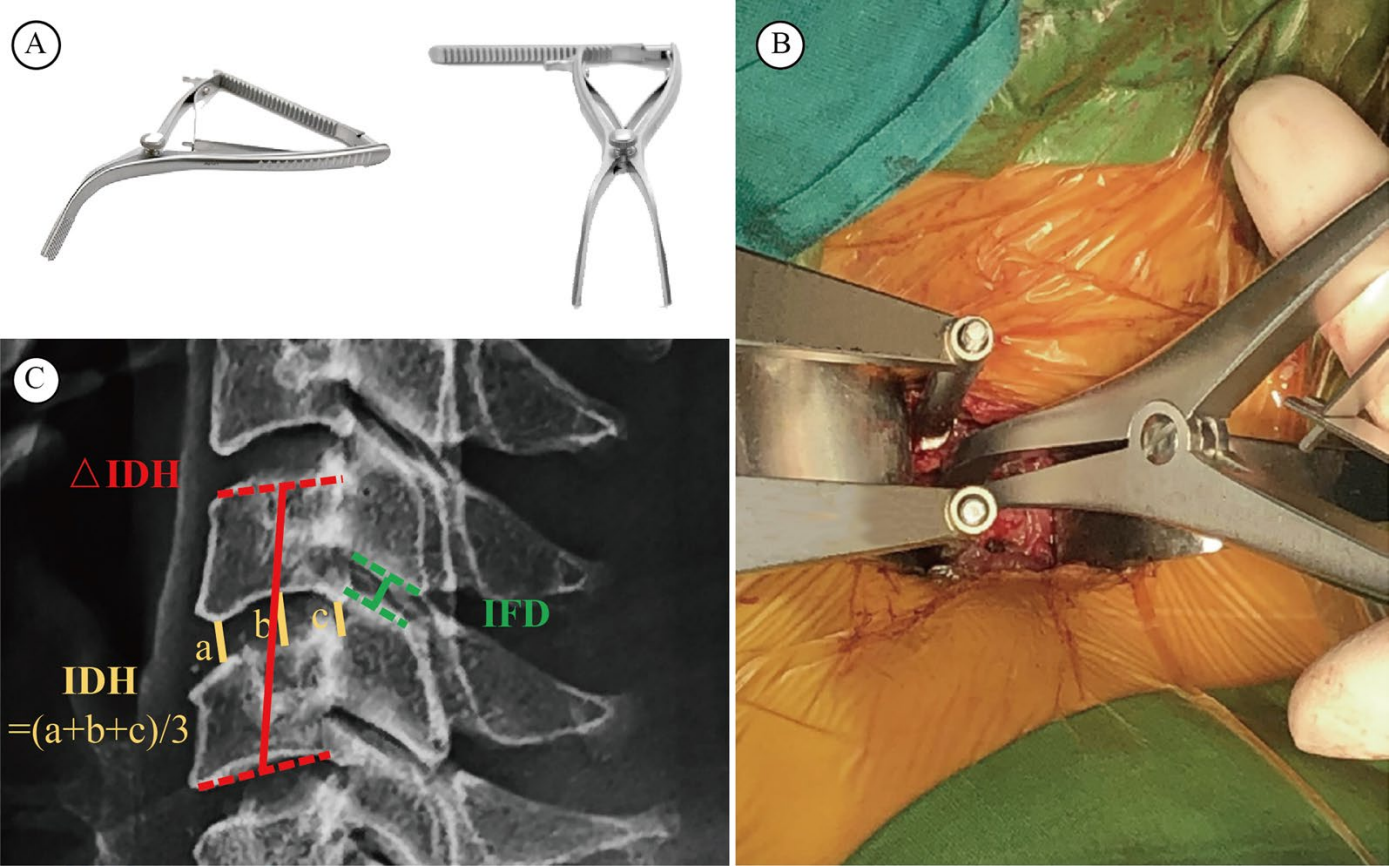
Cervical vertebral spreader and its usage in vertebral distraction. **A** Typical images of vertebral spreader. **B** The Caspar distraction device is first used after removal of the intervertebral disc, and then the vertebral spreader is used to further enlarge the disc space. **C** The illustration showing the measurement method of intervertebral disc height (IDH), change of IDH (△IDH) and interfacet distance (IFD)

For radiological assessment, cervical spine anteroposterior, lateral, flexion, and extension X-ray radiographs were routinely taken preoperatively, 3 days postoperatively, 1-month postoperatively, 2-month postoperatively and 6-month postoperatively. Lateral radiographs of the cervical spine were taken with the patient’s supine head position and horizontal gaze maintained and were used for radiological assessment. The intervertebral disc height (IDH) and interfacet distance (IFD) of the operated segment were measured preoperatively and postoperatively. The IDH was measured using a modified three-point scale method (modified Lane's protocol), and significantly narrowed disc space was defined as a loss of disc height of 50% or more relative to the above normal disc. The increase in postoperative IDH (△IDH) was determined based on the difference between the preoperative and postoperative distances of the fusion segment (Due to decompression manoeuvre, the accuracy of the IDH measurement over the operational segment may be compromised after surgery, so we assessed the mid-point distance between the superior end plate of the upper vertebral body and the inferior end plate of the lower vertebral body on lateral cervical X-ray radiograph as fusion segment height, and △IDH = postoperational fusion segment height—preoperational fusion segment height, Fig. 1). The increase in postoperative IFD (△IFD) was determined based on the difference between the preoperative and postoperative IFD values of the fusion segment (assessed by calculating the mid-point distance between the facet joints of the operated segment on lateral cervical X-ray radiograph, Fig. 1). Measurements were performed by a Centricity PACS 4.0 system (GE Healthcare, USA), and contrast adjustment was made to visualize all vertebrae of the cervical spine. Two independent clinical research assistants who were not involved with the study and were blinded to all clinical information performed radiological measurements, and the average values of both observers were used in the present study.

### Surgical procedures and grouping method

Two surgical groups were distinguished by the distraction techniques, and the patients were blinded and randomly enrolled into each surgical group. The surgical procedure was performed using a standard Smith-Robinson approach to expose the symptomatic level in all the patients. The surgical technique used was basically the same except the distraction procedure in all the patients. In brief, after the removal of the anterior longitudinal ligament and the herniated disc of the involved segment, an additional disc space of 4–6 mm was distracted using a Casper pin distractor system alone (Caspar alone group), or distraction using a Casper-spreader-Casper sequence defined as Casper + spreader group (After a 2–4 mm distraction made by the Casper pin distractor, the intervertebral spreader was inserted into the disc space and further distracted additional 2–3 mm using the spreader, and finally the Casper pin distractor is adjusted to appropriately maintain the enlarged disc space Fig. 1).

Further decompression was performed by removing the superior and inferior end plates, osteophytes, and remnant disc materials. The posterior longitudinal ligament was resected if necessary. For posterolateral decompression, osteophytic overgrowth in the uncovertebral joint was removed to free the nerve roots. A suitable implant was then inserted into the disc space (either the Zero-P system or a traditional cage and plate system, all from Synthes GmbH Switzerland).

### Cervical cadaver study

Four fresh-frozen human cadaveric cervical spines (C2–T1) were used in the biomechanical test. Examinations were first performed to exclude cervical trauma, deformities and other related diseases in these cadavers. Muscular and fascial tissues were removed, and the vertebrae, intervertebral discs, ligaments and facet capsules were entirely preserved. The distal (T1) end of the specimen was embedded in polymethylmethacrylate for stabilization. The facet contact force was tested with force-measuring sensors (Tekscan, Inc., USA). After preparation, the sensors were placed into the facet joint capsule of C3–C4, C4–C5, and C5–C6 in all specimens. Then, the force sensor was calibrated and recorded on the computer during different distraction methods performed on C4–C5. The testing and data collecting was also divided into two groups according to the different distraction method. For Caspar alone group, we continuedly measured the facet joint force change after every 0.5 mm distraction made by the Caspar distractors. For Casper + spreader group, we first made a 2 mm distraction using the Casper distractor, then the intervertebral spreader was inserted into the posterior portion of the vertebrae, and made an additional 2 mm using the spreader, during which the facet joint force changes after every 0.5 mm distraction made by the distractors.

### Statistical analyses

Data analyses were performed using SPSS version 20 for Windows (SPSS, Inc., Chicago, IL, USA). Data are presented as the number of subjects in each group or the mean ± SD. Each independent variable was compared between the two groups using the Mann–Whitney U test for continuous variables and the χ2 test or Fisher exact test for categorical variables. Linear regression analysis was used to identify the relationship between factors and postoperative neck pain. ANOVA and Student’s t test were used to compare the pressure on the facet joint in the cadaver study. A statistically significant difference was set at a *P* value < 0.05.

## Results

### General information of the patients

Between January 2018 and January 2020, 94 consecutive patients (male:female ratio = 40:54) underwent single-level ACDF for cervical spondylotic myelopathy or radiculopathy and completed the 1-year follow-up. The demographic, surgical profile and preoperative cervical alignment data of the patients are summarized in Table 1. The mean age was 57.6 years (ranging from 40 to 75 years). Single-level ACDF was performed at C4−5 in 23 patients, at C5−6 in 56 patients and at C6−7 in 15 patients. The mean disc height was 3.3 ± 1.5 mm preoperatively and increased to 6.4 ± 2.5 mm postoperatively. The mean interfacet distance was 1.9 ± 0.8 mm preoperatively and increased to 2.8 ± 0.7 mm postoperatively. The mean preoperative NDI score was 33.2 ± 9.8 points, the mean preoperative cervical JOA score was 9.4 ± 4.5, the mean preoperative nVAS score was 4.2 ± 3.9, and the preoperative mean aVAS score was 4.9 ± 4.1 (Table 1). All patients had achieved segmental fusion at the 1-year follow-up, and no adjacent segmental degeneration was observed. Satisfactory improvement (improvement rate of more than 60%) in neurological symptoms (NDI scores and JOA scores) was achieved at the 1-year follow-up. nVAS and aVAS scores improved in almost all patients at the 1-year follow-up. Among these patients, 33 patients (35.1%) showed increased neck pain at 3 days after the surgery, 26 patients showed sustained (unrelieved) neck pain at 1 month after the surgery, 21 patients showed sustained (unrelieved) neck pain at 2 months after the surgery, 10 patients had complaints about persistent neck pain at the 6-month follow-up, and 2 patients had persistent postoperative axial pain until 1 year after the surgery.

**Table 1:** Summary of cervical spondylotic myelopathy patient and surgical profile

	Mean ± SD
Number of cases	94
Age (years)	57.6 ± 13.6 (40–75)
Gender (male: female)	40:54
Operative segments	
C4–C5	23
C5–C6	56
C6–C7	15
Preoperative disc height (mm)	3.5 ± 1.3
Preoperative interfacet distance (mm)	1.9 ± 0.8
Preoperative NDI score	33.2 ± 9.8
Preoperative JOA score	9.4 ± 4.5
Preoperative nVAS score	4.2 ± 3.9
Preoperative aVAS score	4.9 ± 4.1

*JOA* Japanese Orthopedic Association for cervical myelopathy, *SD* standard deviation, *nVAS* neck pain Visual Analog Scale, *aVAS* arm pain Visual Analog Scale, *NDI* Neck Disability Index

To determine the possible factors that influence the incidence of postoperative axial pain, we assessed the correlation between preoperative JOA, NDI and arm VAS scores and the neck VAS score at each follow-up time point using linear regression. Additionally, we assessed the correlation between the postoperative change in intervertebral disc height and postoperative change in interfacet distance with the neck VAS score at each follow-up time point. However, the results showed no significant correlation between these factors (Table 2).

**Table 2:** Correlation between clinical parameters and postoperative neck VAS score

	3 days-Postop	1 months-Postop	2 months-Postop	6 months-Postop
△IDH	0.081	0.295	0.438	0.659
△IFD	0.128	0.307	0.585	0.658
JOA	0.231	0.258	0.536	0.842
NDI	0.031	0.052	0.127	0.425
aVAS	0.103	0.231	0.354	0.558

△*IDH* postoperative change of intervertebral disc height, △*IFD* postoperative change of interfacet distance, *JOA* Japanese Orthopedic Association for cervical myelopathy, *nVAS* neck Visual Analog Scale, *aVAS* arm pain Visual Analog Scale, *NDI* Neck Disability Index. A *P* value of less than 0.05 was considered to indicate a statistically significant difference

### Relationship of distraction height and clinical outcome

Since overdistraction is considered a risk factor for postoperative axial pain, we divided the patients into a low △IDH group (*n* = 47) and a high △IDH group (*n* = 47) according to the mean intervertebral space increase of all patients (mean △IDH = 2.73 mm in all patients). By assessing the differences in clinical parameters at each follow-up time point, we found that although NDI scores, JOA scores, and VAS scores for neck and arm pain were much improved, there were no significant differences between the two groups (*p* > 0.05, Table 3).

**Table 3:** Comparison of clinical parameters according to the postoperative intervertebral disc height change

	Low △IDH	High △IDH	*P*
Number of cases	47	47	–
△IDH (mm)	2.4 ± 0.9	3.9 ± 1.0	< 0.01*
3 days-Postop (mean ± SD)			
JOA	9.4 ± 3.8	9.6 ± 4.1	0.532
NDI	29.5 ± 7.2	28.9 ± 8.1	0.374
nVAS	4.4 ± 3.2	4.2 ± 2.8	0.141
aVAS	3.5 ± 2.4	3.4 ± 2.5	0.482
1 months-Postop (mean ± SD)			
JOA	10.6 ± 3.7	10.4 ± 3.9	0.750
NDI	24.2 ± 8.4	23.9 ± 9.1	0.415
nVAS	2.4 ± 1.9	3.0 ± 2.2	0.063
aVAS	3.2 ± 2.2	2.9 ± 2.4	0.129
2 months-Postop (mean ± SD)			
JOA	11.1 ± 2.5	11.5 ± 2.4	0.541
NDI	19.2 ± 7.6	18.8 ± 8.3	0.241
nVAS	1.9 ± 1.5	2.5 ± 1.9	0.084
aVAS	2.7 ± 2.3	2.5 ± 2.2	0.157
6 months-Postop (mean ± SD)			
JOA	12.8 ± 2.9	13.6 ± 3.1	0.221
NDI	13.2 ± 9.2	12.9 ± 8.5	0.185
nVAS	1.7 ± 1.5	1.9 ± 1.4	0.284
aVAS	2.0 ± 1.8	1.9 ± 1.7	0.377

△*IDH* postoperative change of intervertebral disc height, *JOA* Japanese Orthopedic Association for cervical myelopathy, *nVAS* neck Visual Analog scale, *aVAS* arm pain Visual Analog scale, *NDI* Neck Disability Index, *SD* standard deviation. A *P* value of less than 0.05 was considered to indicate a statistically significant difference, and marked with a asterisk *

### Relationship of different distraction methods and clinical outcomes

Next, we evaluated whether the use of an intervertebral spreader affected the clinical outcomes, especially postoperative axial pain. The patients were randomly divided into two groups: the Caspar alone group (*n* = 47) and Caspar + spreader group (*n* = 47) group. Although no significant differences were found in the cervical JOA or aVAS scores between the groups, the nVAS and NDI scores showed significant differences in the early follow-up period (Table 4). VAS scores for neck pain were lower in the Caspar + spreader group than in the Caspar alone group at the 3-day, 1-month and 2-month follow-ups (*p* = 0.011, 0.021, and 0.042, respectively). The NDI was significantly lower in the Caspar + spreader group than in the Caspar alone group at 3 days post operation but showed no significant difference at the later follow-ups. These results showed that the intervertebral-spreader-assisted distraction method could indeed alleviate postoperative axial pain.

**Table 4:** Comparison of clinical parameters according to the different vertebral distraction method

	Caspar alone	Caspar + Spreader	*P*
Number of cases	47	47	–
3 days-Postop (mean ± SD)			
JOA	9.3 ± 4.0	9.2 ± 3.8	0.389
NDI	31.5 ± 7.2	27.9 ± 7.8	0.048*
nVAS	4.4 ± 3.3	2.4 ± 1.8	0.011*
aVAS	3.2 ± 2.5	3.1 ± 2.6	0.382
1 months-Postop (mean ± SD)			
JOA	10.2 ± 3.4	10.7 ± 4.1	0.364
NDI	25.2 ± 8.4	23.9 ± 9.1	0.213
nVAS	3.9 ± 2.4	1.9 ± 1.2	0.021*
aVAS	3.0 ± 2.3	2.8 ± 2.5	0.182
2 months-Postop (mean ± SD)			
JOA	11.1 ± 2.5	12.1 ± 2.6	0.541
NDI	19.2 ± 7.6	18.8 ± 8.3	0.241
nVAS	2.9 ± 2.0	1.3 ± 0.6	0.042*
aVAS	2.6 ± 2.4	2.5 ± 2.2	0.357
6 months-Postop (mean ± SD)			
JOA	12.9 ± 2.8	13.8 ± 3.0	0.146
NDI	12.5 ± 9.7	10.5 ± 9.5	0.105
nVAS	1.8 ± 1.7	1.1 ± 0.5	0.142
aVAS	2.0 ± 1.8	1.9 ± 1.6	0.377

*JOA* Japanese Orthopedic Association for cervical myelopathy, *nVAS* neck Visual Analog scale, *aVAS* arm pain Visual Analog scale, *NDI* Neck Disability Index, *SD* standard deviation. A *P* value of less than 0.05 was considered to indicate a statistically significant difference, and marked with a asterisk *

### Facet joint pressure comparison between different distraction methods

**Fig. 2:**
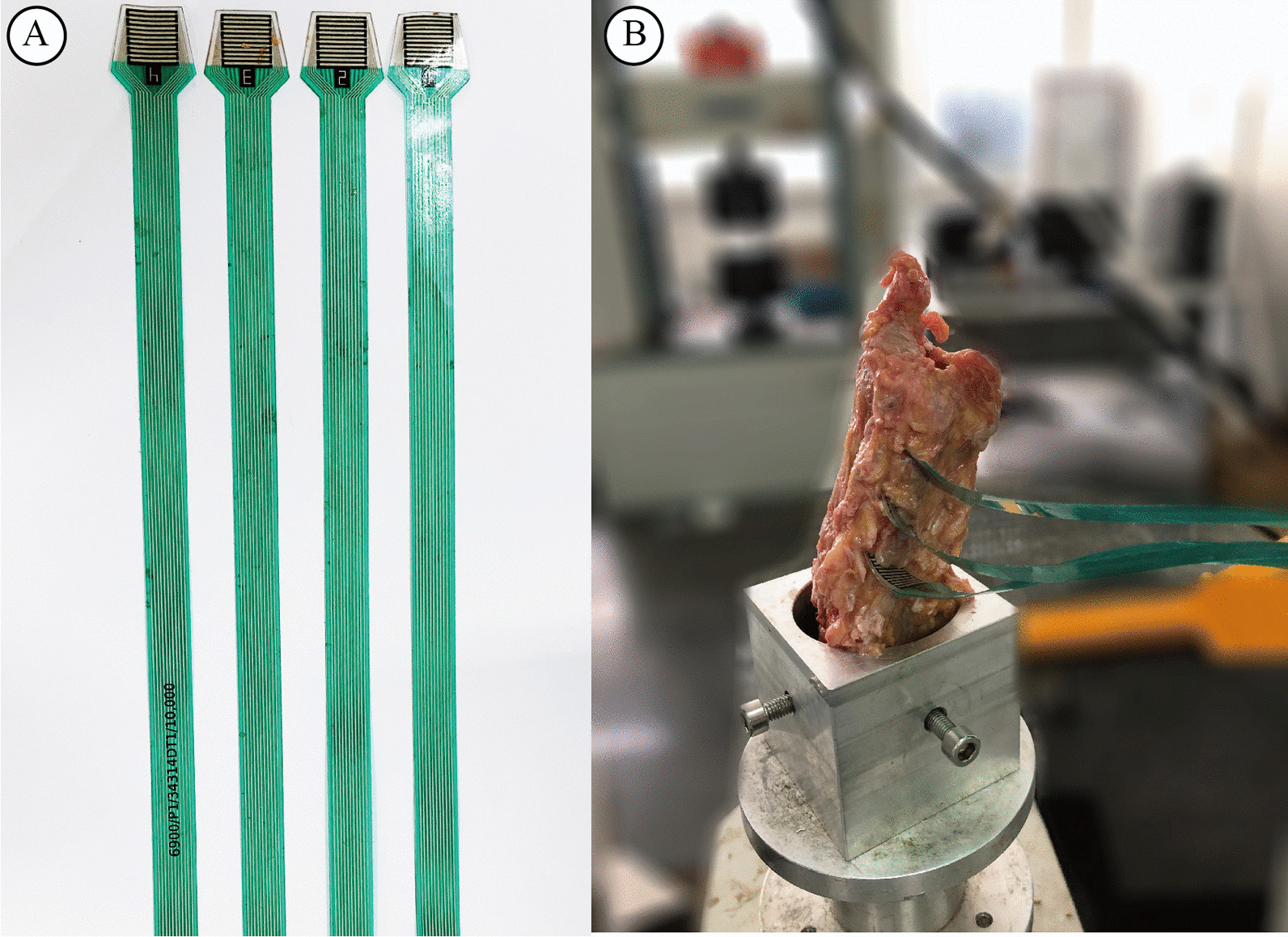
Images of pressure sensor and cervical cadaver model to measure the facet joint pressure during distraction. **A** Typical images of pressure sensor used during the facet joint force and pressure measuring. **B** Four cervical spine cadavers were used to measure the facet joint force and pressure during distraction, the coordinate facet joint and its upper and lower facet joints were measured simultaneously

**Fig. 3:**
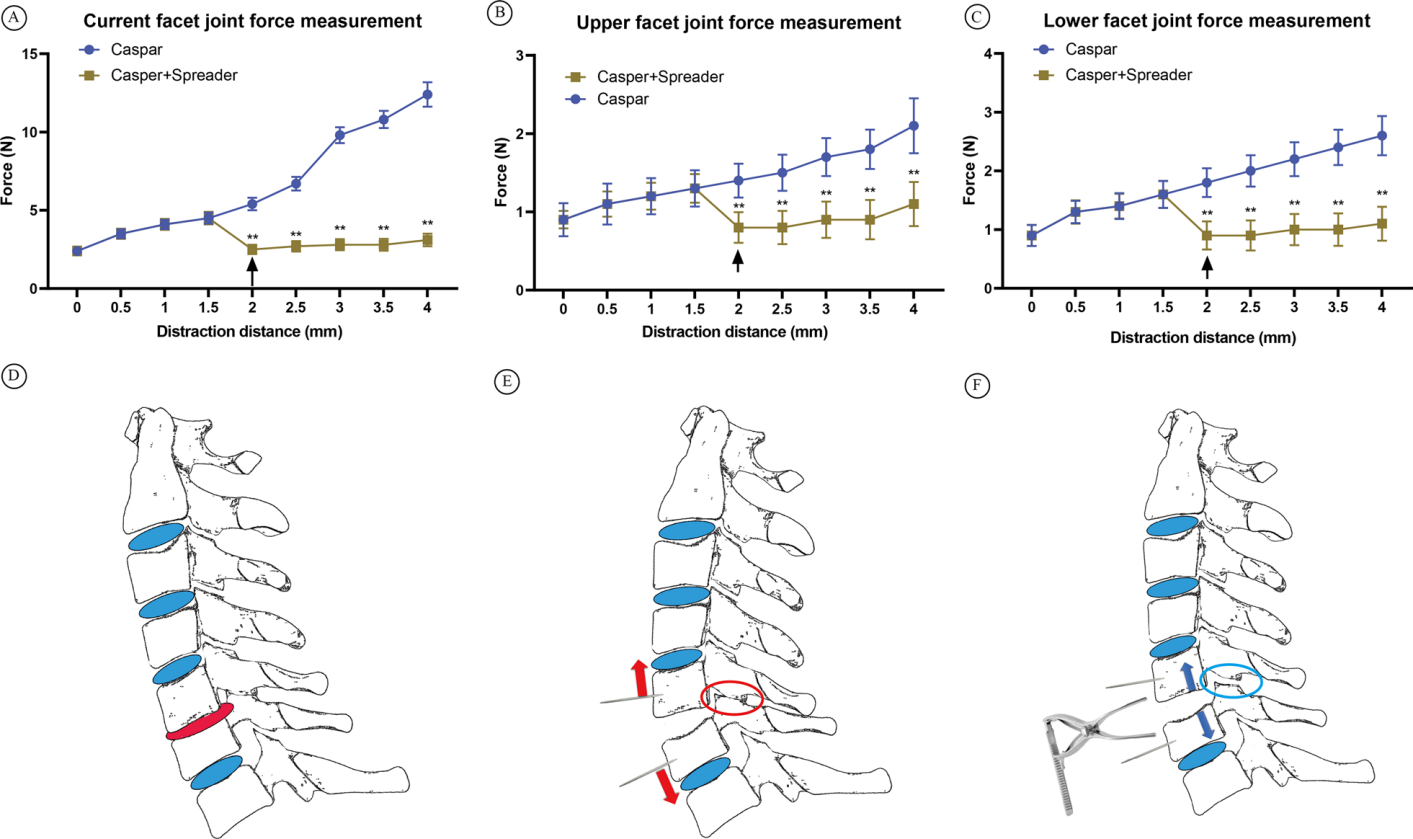
The differences of facet joint forces during different distraction method. The facet joint pressure is measured and recorded during the vertebral distraction procedure at every 0.5 mm increase using the distraction device. The change of current facet joint (**A**), the upper-level facet joint (**B**), and the lower-level facet joint (**C**) is compared using two distraction method. The arrow indicates the time point when intervertebral spreader takes place, and from the arrow indicated distraction distance on, only the spreader is used to distract the vertebrates. Illustrations showing that when degeneration occurs, the involved disc is flattened and herniated, causing the intervertebral space and facet joint space narrowed (**D**). **E** Traditional distraction using Casper system concentrates its distraction force on vertebral pins, which is at the front of the segment, and can cause uneven distraction that result in further narrowing of the facet joint. **F** The use of intervertebral spreader concentrates the distraction force at the posterior margin of the endplate, which cause axial distraction force to restore the facet joint structure, and also lowered the pressure in facet joints

Since a report showed that facet joint pressure changes may cause postoperative neck pain, we assessed the facet joint pressure in cervical cadaver spinal segments using different distraction methods. To mimic the ACDF procedure, we first removed the C4/5 intervertebral disc and then placed the sensors into the facet joints of C3/4, C4/5 and C5/6 (Fig. 2). By comparing the facet joint force and pressure change in the two distraction methods, we found that the facet joint pressure was significantly lowered when an intervertebral spreader was used to further distract the vertebrae (Fig. 3, Table 5). Moreover, aside from the current facet joint, the upper and lower adjacent facet joints all significantly alleviated the force and pressure increase caused by Casper distraction (Fig. 3, Table 5). The results showed that the use of an intervertebral spreader alleviated almost half of the joint pressure increase after Casper pin distraction, not only to its current facet joint but also to the adjacent joints.

**Table 5:** Comparison of facet joint pressure after distraction on cervical spine cadaver (3 mm of distraction)

Facet joint	Caspar alone (mean ± SD)		Caspar + spreader (mean ± SD)		*P* (Force)	*P* (Pressure)
	△Force (N)	△Pressure (N/cm^2^)	△Force(N)	△Pressure (N/cm^2^)		
Upper level	2.1 ± 0.5	1.5 ± 0.2	0.8 ± 0.2	0.5 ± 0.1	0.01*	0.01*
Current level	9.8 ± 0.7	7.2 ± 0.5	2.7 ± 0.6	1.8 ± 0.4	0.01*	0.01*
Lower level	2.2 ± 0.4	1.6 ± 0.3	1.0 ± 0.2	0.6 ± 0.2	0.01*	0.01*

△*Force* change of facet joint force measured by sensors, △*Pressure* change of facet joint pressure measured by sensors, *Current level* the coordinate facet joint of vertebral distraction, *Upper level* the upper facet joint of vertebral distraction, *Lower level* the lower facet joint of vertebral distraction, *SD* standard deviation. A *P* value of less than 0.05 was considered to indicate a statistically significant difference, and marked with a asterisk *

## Discussion

Axial neck pain is a common complaint after ACDF during the follow-up period [20]. Axial neck pain results in a high economic burden and a series of negative effects on patients, including effects on their work, social life and treatment [21]. Zhou et al. [20] reported that the incidence of postoperative axial pain after ACDF was up to 29.91%. According to Kawakami et al. [11], nearly 38.3% of ACDF patients suffer from axial neck pain during the follow-up period. Another study produced by Ylinen et al. [22] indicated that approximately forty-three percent of patients experienced moderate or high axial neck pain and that neck movement and strength decreased after ACDF operation. In our study, we found that more than 30% of single-level ACDF patients suffered from postoperative axial pain. Although axial pain is commonly reduced after 6 months, early postoperative axial pain can significantly affect the patients’ initial clinical outcome after surgery and is a prominent issue that needs to be resolved. In our study, we found that using a different distraction method could significantly affect the initial neck VAS scores of patients and may be a possible way to lower the incidence of postoperative axial pain in ACDF-treated patients.

ACDF, as the most effective anterior approach, is widely used for the standard surgical treatment of cervical spondylosis. In the conventional ACDF procedure, vertebral distraction is the routine and vital method performed by vertebral distractors to further expose the intervertebral space to achieve complete decompression of the cord and nerve roots. The most common distractor used was the Caspar retractor system, which is based on distraction through pins fixed on vertebral bodies [18]. However, reports have shown that improper distraction can lead to postoperative axial pain due to damaged or overpressurized facet joints [16, 23–25]. The latest study based on the three-dimensional finite element model indicated that facet joint pressure increased after ACDF operation and was aggravated when moving [26]. This may be related to damage to the stable biological structure, which consists of facet joints and intervertebral discs in the spinal segment, during vertebral distraction. The Caspar device distracts the two vertebral bodies using a biased axial force, which leads to an imbalance (or possibly angular imbalance) between the anterior column and posterior column. Moreover, the torques of the vertebral body to the facet joints are obviously increased. Ha et al. [16] indicated that excessive vertebral distraction obviously caused mechanical overload on facet joints and recommended that the torque be less than 6 kgf·cm during ACDF. Bai et al. [12] published a retrospective review that found that the incidence of postoperative axial pain significantly increased if the percentage change in the intervertebral height of the operated segment after surgery was over 10%. Chang et al. [15] demonstrated that the facet force significantly improved in extension mode under a maximum torque of 2 Nm in ACDF-treated spines by measuring the force of the adjacent-level facet joint in eighteen cadaveric cervical spines. Thus, how to properly distract the vertebrae and avoid postoperative axial pain is essential to the clinical outcomes of cervical spondylosis patients who undergo ACDF treatment.

The intervertebral spreader was designed to spread the attached bones and is used in many stages. However, it was first used to spread the significantly narrowed disc space to open up enough space for decompression procedures in our institute. During practice, we found that the spreader distracts the vertebral bodies by concentrating the axial force on the posterior margin of the upper and lower end plates, which is more central to the middle column of the spine than with Casper distractors. This may lead to the maintenance of the biological structure by not angularizing the segments and causing pressure on facet joints (Fig. 3). Based on this hypothesis, we performed a clinical retrospective evaluation and found that the use of intervertebral spreaders can indeed lead to better outcomes, especially in postoperative axial pain, in single-level ACDF patients. Through a cadaver study, we confirmed the alleviation of facet joint pressure with the use of intervertebral spreaders.

Postoperative axial pain affects patients’ surgical outcomes, but there is still no effective way to fully avoid it, and the mechanism through which it occurs is still unclear. Zhou et al. [20] analysed the preoperative risk of axial pain after ACDF and indicated that its incidence in a segmental kyphosis group was 2.9 times higher than that in a segmental lordosis group. The reason for this may be due to the imbalance of the neck muscles and the facet joints. By measuring SVA, global lordotic angle and other indicators, Kirzner et al. [27] demonstrated that the sagittal balance of the lateral view of the cervical spine was important to postoperative axial pain. Numerous studies have suggested that the facet joint is the main postoperative axial pain generator. Ha et al. [16] found that overdistraction may damage the facet joint, which will eventually lead to postoperative axial pain. According to a review by Cavanaugh et al. [28], changes in the facet joint play an extremely important role in axial pain. The facet joint capsule contains not only peripheral nerves but also mechanical and nociceptive receptors, which could cause severe pain when stimulated [29]. However, more precise evidence is needed to confirm that postoperative axial pain is directly related to the facet joint or other tissue in the posterior column of the cervical spine.

Although the results in our study support that the use of intervertebral spreaders may lower the incidence of postoperative axial pain, there are still limitations to this study. First, the biomechanical experiments were carried out only on cadaveric cervical spine specimens because they cannot be performed during operations on living patients; therefore, the effect of muscles were not taken into consideration. Second, this was a retrospective analysis of patients at a single institution; therefore, there is a risk of selection bias. Furthermore, the precise mechanism by which the pressure on the facet joint affects postoperative axial neck pain is far from being fully revealed. Last, the exact normal value of the height of the posterior vertebral distraction is still not clear. We hope further in-depth clinical and biomechanical studies will be carried out to further explain the relationship between vertebral distraction and axial pain.

## Conclusions

This study describes that vertebral distraction using a Casper device significantly increased the load on facet joints, and the use of an intervertebral spreader could largely alleviate the pressure and lower the incidence of postoperative axial neck pain in single-level ACDF-treated patients. These results provide evidence supporting the use of a modified ACDF vertebral distraction procedure to lower the incidence of postoperative axial pain.

## Data Availability

Data sharing is not applicable to this article as no datasets were generated or analysed during the current study.

## Abbreviations

ACDF: Anterior cervical discectomy and fusion
ASD: Adjacent segment degeneration
Zero-P: Zero-profile
JOA: Japanese Orthopedic Association score
VAS: Visual Analog Scale
ROM: Range of motion
